# Ellis-Van Creveld syndrome

**DOI:** 10.1186/1750-1172-2-27

**Published:** 2007-06-04

**Authors:** Geneviève Baujat, Martine Le Merrer

**Affiliations:** 1 Centre de Référence des Maladies Osseuses Constitutionnelles, Hôpital Necker-Enfants Malades, 149 rue de Sèvres 75743, Paris Cedex 15, France

## Abstract

Ellis-van Creveld syndrome (EVC) is a chondral and ectodermal dysplasia characterized by short ribs, polydactyly, growth retardation, and ectodermal and heart defects. It is a rare disease with approximately 150 cases reported worldwide. The exact prevalence is unknown, but the syndrome seems more common among the Amish community. Prenatal abnormalities (that may be detected by ultrasound examination) include narrow thorax, shortening of long bones, hexadactyly and cardiac defects. After birth, cardinal features are short stature, short ribs, polydactyly, and dysplastic fingernails and teeth. Heart defects, especially abnormalities of atrial septation, occur in about 60% of cases. Cognitive and motor development is normal. This rare condition is inherited as an autosomal recessive trait with variable expression. Mutations of the *EVC1 *and *EVC2 *genes, located in a head to head configuration on chromosome 4p16, have been identified as causative. EVC belongs to the short rib-polydactyly group (SRP) and these SRPs, especially type III (Verma-Naumoff syndrome), are discussed in the prenatal differential diagnosis. Postnatally, the essential differential diagnoses include Jeune dystrophy, McKusick-Kaufman syndrome and Weyers syndrome. The management of EVC is multidisciplinary. Management during the neonatal period is mostly symptomatic, involving treatment of the respiratory distress due to narrow chest and heart failure. Orthopedic follow-up is required to manage the bones deformities. Professional dental care should be considered for management of the oral manifestations. Prognosis is linked to the respiratory difficulties in the first months of life due to thoracic narrowness and possible heart defects. Prognosis of the final body height is difficult to predict.

## Disease name

Ellis-van Creveld syndrome (MIM 225500)

Chondroectodermal dysplasia

Mesoectodermal dysplasia

## Definition

Ellis van Creveld syndrome (EVC) is an autosomal recessive skeletal dysplasia, with inter- and intra-familial variability, characterized by short ribs, short limbs, postaxial polydactyly, and dysplastic teeth and nails. Congenital heart defects occur in 60% of the individuals. Mutations in the *EVC1 *and *EVC2 *genes are associated with this syndrome.

## Epidemiology

EVC is a rare disease. The exact prevalence remains unknown. About 100 cases have been reported between the first full description of the syndrome in 1940, by R. Ellis and S. Van Crefeld [1], and 1968 [2]. Since 1968, approximately 50 other cases have been reported in the literature. EVC is found with an increased frequency among the Amish community in Lancaster Country, Pennsylvania, US, where the largest pedigree has been described: 52 cases in 30 sib ships [3].

## Clinical description

EVC phenotype is variable and affects multiple organs. General detailed descriptions of the clinical manifestations in limited series or single reports are available [3-10].

Prenatal abnormalities may be early discovered, after the 18^th ^gestation week; they include narrow thorax, marked shortening of the long bones, hexadactyly of hands and feet, and cardiac defect [11,12], leading to discussing the diagnosis of short rib-polydactyly syndromes (cf. below). Increased first-trimester fetal nuchal translucency thickness in association with ECV has been described at 13^th ^week of gestation [12].

**Figure 1:**
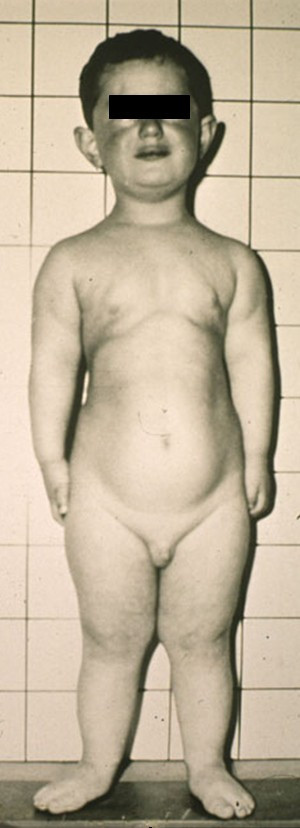
Patient at the age of 5 years showing long narrow chest and shortness of the limbs.

**Figure 2:**
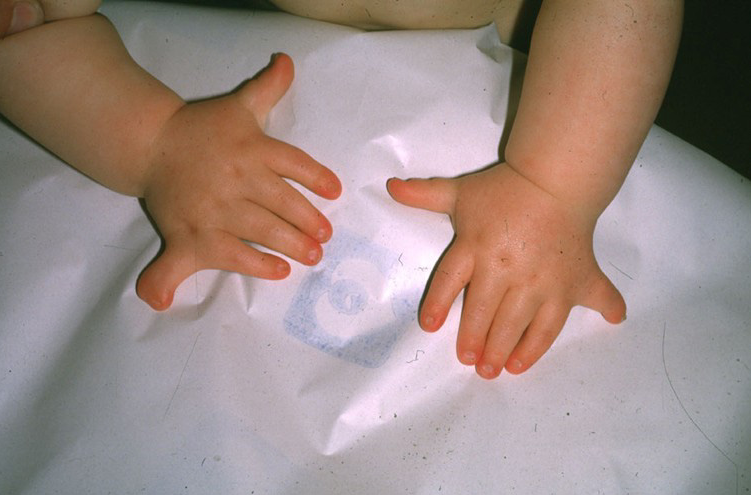
Bilateral polydactyly with short fingers in EVC patient.

**Figure 3:**
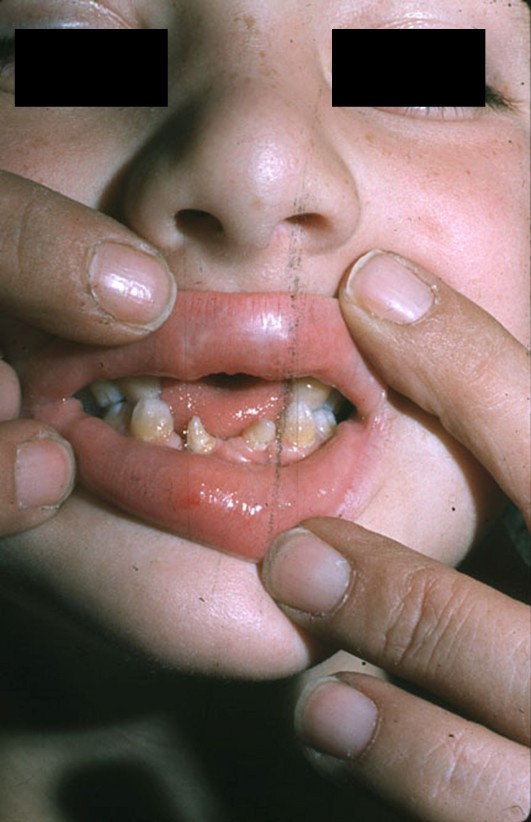
Anterior view of the mouth of EVC patient showing absence of upper incisors and conical lower incisors.

After birth, the cardinal features usually present are: 1) disproportionate small stature whith increasing severity from the proximal to distal portions of the limbs, and shortening of the middle and distal phalanges (Figure 1); 2) polydactyly affecting hands (uni [exceptional: [13]] – or, usual, bilateral) and, occasionally, the feet (Figure 2); 3) hidrotic ectodermal dysplasia mainly affecting the nails, hair and teeth; 4) congenital heart malformations occurring in about 50–60% of cases and comprising of single atrium, defects of the mitral and tricuspid valves, patent ductus, ventricular septal defect, atrial septal defect and hypoplastic left heart syndrome. The presence of congenital heart disease may support the diagnosis of the EVC syndrome and appears to be the main determinant of longevity [6,14].

The oral manifestations spectrum is wide, including malocclusion, labiogingival adherences and gingival hypertrophy, labiogingival frenulum hypertrophy, accessory labiogingival frenula, serrated incisal margins, dental transposition, diastrema, conical teeth, enamel hypoplasia and hypodontia (Figure 3). Teeth may be prematurely erupted, at birth, or exfolliate prematurely [15].

Several inconstant additional clinical findings are described, including strabismus, epi- and hypospadias, cryptorchidism [3], and thoracic wall and pulmonary malformations [16]. Renal abnormalities are found in very rare cases with agenesis, dysplasia, megaureter and nephrocalcinosis. Lethal nephronophthisis has been reported only once, in a patient with short limbs, short ribs, abnormal teeth and polydactyly (considered as EVC) [17], but this diagnosis may be discussed. Hematologic abnormalities have also been rarely reported: one case with dyserythropoiesis [18] and another associated with perinatal myeloblastic leukemia [19]. Head circumference and mental developement in EVC are normal.

Because of the overlap between EVC and other short ribs-chondrodysplasias, and because molecular studies are only recently available and non-constantly performed, some old reports of EVC associated with exceptional features should be carefully read, since they may be mixed with publications that evidently do not deal with the EVC syndrome.

The possibility of manifestations in heterozygous carriers has been discussed for a long time. From the observation of the large Amish kindred, McKusick concluded that there is no heterozygous manifestation of EVC [3]. However, other authors described polydactyly in relatives of four unrelated EVC families [20,21]. Spranger and Tariverdian [22] described a father of an EVC patient with fingers and teeth abnormalities, and then reviewed other reports of possible heterozygous manifestations. The Weyers acrofacial dysostosis, an autosomal dominant disorder described in 1952, is characterized by variable extremities and facial features. This condition has been found to be associated with *EVC *and *EVC2 *mutations that, finally, confirmed that Weyers dysostosis represents the heterozygous expression of the mutation, which, in homozygous form, causes the autosomal recessive disorder EVC [23,24].

### Skeletal features

A variety of radiological skeletal features may be observed, including retarded bone maturation, fusion of the hamate and capitate bones of the wrist, defect of the lateral aspect of the proximal part of the tibia (knock-knees), cubitus valgus, hypoplastic cubitus, supernumerary carpal bone centre, clinodactyly of the 5^th ^finger, fusion of the 5^th ^and 6^th ^metacarpals, disturbance in bone modeling of the metacarpals and/or phalanges. Bone age is usually retarded.

## Etiology

The *EVC *gene was previously localized by linkage analysis to the distal short arm of chromosome 4 [25,26], in an area proximal to other chondrodystrophias. Mutations in this gene have been identified in EVC individuals from the Amish cases and from other pedigrees (Mexico, Equador and Brazil) [23]. But the screening of the 21 *EVC *coding exons in 58 patients with EVC permitted to identify only 13 patients with homozygous mutations. Among the remaining 45 cases, no mutation in one or two allele was identified [27]. The cDNA analysis of fibroblast RNA from three of these patients was also normal. These observations had raised the possibility of genetic heterogeneity. A second gene, *EVC2*, was identified in an Ashkenasi child, immediately adjacent to *EVC *(named *EVC1 *or *EVC*), in a head to head configuration [28]. Expression of the gene is found in heart, placenta, lung, liver and skeletal muscle. *EVC2 *spans 166.4 kb and shares a common promoter region with *EVC*. The transcriptional start sites of *EVC *and *EVC2 *are separated by only 1643 bp. There is no significant sequence homology between *EVC *and *EVC2 *at either protein or nucleic levels. The *EVC2 *gene encodes a protein predicted to have one transmembrane segment, three coiled-coil regions, and one RhoGEF domain (SMART). The EVC2 protein has significant sequence homology with the tail domains of class IX non muscle myosins (BLAST). Mutations in the *LIMBIN *gene, a bovine orthologue of *EVC2*, are associated with bovine chondrodysplastic dwarfism [28]. Affected EVC individuals with mutations in *EVC *or *EVC2 *are phenotypically indistinguishable [27]. An interesting recent work with sequencing *EVC *and *EVC2 *in a series of 65 EVC patients identified *EVC *mutations in 20 families and *EVC2 *mutations in 25 families [29]. No mutation has been identified in 20/65 families but as none of these individuals had consanguineous parents (even if it was possible that some of these cases were misdiagnosed), this has lead to evidence for a genetic heterogeneity in EVC syndrome. As previously mentioned, heterozygous mutations in the *EVC *or *EVC2 *genes also cause Weyers acrofacial dysostosis, an allelic disorder, showing autosomal dominant inheritance.

## Diagnostic methods

The clinical diagnosis is based on observation of the symptoms and manifestations described above and supported by the skeletal survey. The definitive diagnosis is molecular, based on homozygosity for a mutation in the *EVC *and *EVC2 *genes by direct sequencing.

## Differential diagnosis

EVC belongs to the short rib-polydactyly group (SRP). These SRPs are all autosomal recessive disorders that have been classified into types (Saldino-Noonan syndrome, type I; Majewski syndrome, type II; Verma-Naumoff syndrome, type III; Beemer-Langer syndrome, type IV; and Jeune Dystrophy). They are characterized by hypoplastic thorax due to short ribs, short limbs, frequent polydactyly and visceral abnormalities, and are discussed prenatally. Radiographically and histologically, SRP III (Verma-Naumoff syndrome, OMIM 263510) most resembles some forms of EVC [30,31]. The question of SRP being due to mutation in the *EVC1 *gene was excluded by Takamine *et al*. [32].

Postnatally, the essential differential diagnoses include Jeune dystrophy, McKusick-Kaufman syndrome and Weyers syndrome. Jeune dystrophy (MIM 208500) is characterized by thoracic dystrophy, shortening of the extremities and generalized bone dysplasia. Similarities and differences of patients with EVC and Jeune dystrophy have been stressed [33,34]. There are no specific constant features to confirm the diagnosis of presumptive EVC but some features, including congenital heart disease, supernumerary digits and ectodermal dysplasia will mostly support the diagnosis of EVC syndrome than Jeune dystrophy. EVC and McKusick-Kaufman syndrome (MKK, MIM 236700), both recessively inherited disorders, share postaxial polydactyly and congenital heart defect. Distinguishing characteristics are the osteochondrodysplasia and ectodermal anomalies in EVC syndrome, and hydro metrocolpos in MKK syndrome. MKK is caused by mutations in a gene on chromosome 20p12, encoding a protein similar to members of the chaperonin family. Mutation in the same gene causes Bardet-Biedl syndrome-6 [35]. Weyers acrodental dysostosis (OMIM 193530) is, as mentioned above, the heterozygotous manifestation of the *EVC *gene. Disproportionate dwarfism, heart defect and thoracic dysplasia are not present in this autosomal dominant condition.

## Genetic counseling

EVC syndrome is an autosomal recessive disorder, with a mendelian risk of 25% for subsequent pregnancies.

## Prenatal diagnosis

EVC may be detected prenatally by ultrasound examination. The association of several structural fetal defects in the late first trimester, including narrow thorax, short and bowed long bones, rounded metaphyses, postaxial polydactyly, and cardiac defect may suggest the diagnosis ECV. Increased nuchal translucency thickness has been described associated with ECV [12]. In case of potential recurrence, prenatal diagnosis using molecular genetic techniques is, in theory, feasible, on DNA extracted from chorionic villus samples.

## Management

The management of EVC is multidisciplinary. Symptomatic management is mostly required in the neonatal period, including treatment of the respiratory distress due both to narrow chest and heart failure. Neonatal teeth should be removed because they may impair the feeding. In infancy and early adulthood, general and specialized pediatric follow-up are also required: the short stature is considered resulting of chondrodysplasia of the legs and the possible treatment with growth hormone is considered ineffective. It is important to notice, however, that the association of growth hormone deficiency and ECV has been reported in one patient and, in this case, the growth hormone treatment had a favorable effect on growth [36]. The possibility of bones deformity, especially knee valgus with depression of the lateral tibial plateau and dislocation of the patella [37], needs regular orthopedic follow-up. Dentists play an important role in control of dental and oral manifestations. Dental treatment must be performed under prophylactic antibiotic coverage with consideration for the high incidence of cardiac defects in EVC patients. In addition, as in other chondrodysplasias, rare narrowing of the spinal canal can occur and justify a regular follow-up during adult life.

## Prognosis

Although there is no systematic follow-up EVC series reported, the prognosis is linked to the respiratory difficulties in the first months of life and these difficulties are related to thoracic narrowness and possible heart defect.

We insist on the fact that the cognitive development in EVC syndrome is normal. Prognosis of the final body height in individual patients with EVC is difficult to predict, as the rare publications of adult EVC cases report a variable final stature, from 119 cm [8] to 161 cm [4].

## Unresolved questions

### Clinical features

The clinical spectrum of EVC syndrome is not well delineated at present. There are no specific constant features in EVC syndrome: some of them are usually present but their absence does not exclude the diagnosis. Depending on the diagnostic criteria, some patients may be recognized as EVC and other patients not. In the literature, there are many cases described as EVC syndrome in which the diagnosis is uncertain, and some of them definitely represent other entities. The reported data are limited to small series. Careful collection of case history, genotype-phenotype correlation with the two known genes and patient follow-up will provide supplementary information and further delineation of the EVC syndrome, and distinction from the other short rib-polydactyly syndromes.

### Molecular mechanism

ECV is associated with a genetic heterogeneity and *EVC1 *and *EVC2 *do not account for the totality of EVC cases. Further studies are needed to elucidate other genes involved in EVC manifestations. They could also contribute to unraveling specific molecular processes that lead to the phenotypic manifestations of ECV.

## References

[B1] Ellis RW, van Crefeld S (1940). A syndrome characterized by ectodermaldysplasia, polydactyly, chondrodysplasia and congenital morbus cardia. Arch Dis Child.

[B2] Lynch JI, Perry LW, Takakvwa T, Scott LP (1968). Congenital heart disease and chondroextodermal dysplasia. Am J Dis Child.

[B3] Mac Kusick V (2000). Ellis-van Crefeld syndrome and the Amish. Nature Genet.

[B4] Oliveira da Siva E, Janovitz D, Cavalcanti de Albuquerque S (1980). Ellis-van Creveld syndrome: report of 15 cases in an inbred kindred. J Med Genet.

[B5] Waldrigues A, Grohmann L, Takahashi T, Reis H (1977). Ellis van Creveld. An inbred kindred with 5 cases. Rev Bras de Pesquisas Med e Biol.

[B6] Blackburn M, Belliveau R (1971). Ellis-vanCreveld syndrome: a report of previously undescribed anomalies in two siblings. Am J Dis Child.

[B7] Prabhu S, Daftary D, Dholakia H (1978). Chondroectodermal dysplasia (Ellis-van Creveld syndrome): report of 2 cases. J Oral Surgery.

[B8] Renier JC, Larget-Piet L, Boasson M, Berthelot J, Fouillet L (1975). Dysplasie chondroépidermique d'Ellis-van Creveld: deux cas dans une même fratrie. Revue du rhumatisme.

[B9] Goor M, Farriaux J, Dupuis C, François P, Fontaine G (1970). Le syndrome d'Ellis-van Creveld: étude d'une observation familiale. La revue de Pédiatrie.

[B10] Kushnick T, Paya K, Namunes P (1962). Chondroectodermal Dysplasia. Am J Dis Child.

[B11] Horigome H, Hamada H, Sohda S, Oyake Y, Kurosaki Y (1997). Prenatal ultrasonic diagnosis of a case of Ellis-van Creveld syndrome with a single atrium. Pediatr Radiol.

[B12] Venkat-Raman N, Sebire N, Murphy K (2005). Increased first-trimester fetal nuchal translucenty thickness in association with chondroectodermal dysplasia (Ellis-van Creveld). Ultrasound Obstet Gynecol.

[B13] Engle M, Ehlers K (1969). Ellis-van Creveld syndrome with asymmetric polydactyly and successful surgical correction of common atrium. Birth defects Orig Art Ser.

[B14] Digilio M, Marino B, Ammirati A, Borgaza U, Giannotti A, Dallapiccola B (1999). Cardiac malformations in patients with oral-facial-skeletal syndromes: clinical similarities with heterotaxia. Am J Med Genet.

[B15] Winter G, Geddes M (1967). Oral manifestations of chondroectodermal dysplasia (Ellis-Van Creveld Syndrome). Report of a case. Br Dent J.

[B16] Moore T (1963). Chondroectodermal dysplasia (Ellis-van Creveld syndrome) with bronchial malformation and neonatal tension lobar emphysema. J Thoracic and Cardiovas Surg.

[B17] Moudgil A, Bagga A, Kamil ES, Rimoin DL, Lachman RS, Cohen AH, Jordan SC (1998). Nephronophtisis associated with Ellis-van Creveld syndrome. Pediatr Nephrol.

[B18] Scurlock D, Ostler D, Nguyen A, Wahed A (2005). Ellis-van Creveld syndrome and dyserythropoiesis. Arch Pathol Lab Med.

[B19] Miller D, Newstead G, Young L (1969). Perinatal leukemia with a possible variant of the Ellis-van Crefeld. J Pediatr.

[B20] Fryns JP (1991). Postaxial polydactyly as heterozygote manifestation in Ellis-van Creveld syndrome? [letter]. Am J Med Genet.

[B21] Goldblatt J, Minutillo C, Pemberton P, Hurst J (1992). Ellis-van Creveld syndrome in a western Australian Aboriginal community: postaxial polydactyly as a heterozygous manifestation?. Med J Aust.

[B22] Spranger S, Tariverdian G (1995). Symtomatic heterozygozygosity in the Ellis-van Creveld syndrome?. Clin Genet.

[B23] Ruiz-Perez VL, Ide SE, Strom TM, Lorenz B, Wilson D, Woods K, King L, Francomano C, Freisinger P, Spranger S, Marino B, Dallapiccola B, Wright M, Meitinger T, Polymeropoulos MH, Goodship J (2000). Mutations in a new gene in Ellis-van Creveld syndrome and Weyers acrodental dysostosis. Nature Genet.

[B24] Ye X, Song G, Fan M, Shi L, Jabs EW, Huang S, Guo R, Bian Z (2006). A novel heterozygous deletion in the EVC2 gene causes Weyers acrofacial dysostosis. Hum Genet.

[B25] Francomano C, Ortez deLuna R, Ide S, Pyeritz R, Wright M, Polymeropoulos M (1995). The gene for the Ellis-van Creveld syndrome maps to chromosome 4p16. Am J Hum Genet.

[B26] Polymeropoulos M, Ide S, Wright M, Goodship J, Weissenbach J, Pyeritz R, Da Silva E, Ortiz De Luna R, Francomano CA (1996). The gene for the Ellis-van Creveld syndrome is located on chromosome 4p16. Genomics.

[B27] Ruiz-Perez V, Tompson S, Blair H, Espinoza-Valdez C, Lapunzina P, Silva E, Hamel B, Gibbs J, Young I, Wright M, Goodship J (2003). Mutations in two nonhomologous genes in a head-to-head configuration cause Ellis-van Creveld syndrome. Am J Hum Genet.

[B28] Galdzicka M, Patnala S, Hirshman M, Cai J, Nitowsky, Egeland J, Ginns E (2002). A new gene, EVC2, is mutated in Ellis-van Creveld syndrome. Molec Genet Metab.

[B29] Tompson SW, Ruiz-Perez VL, Blair HJ, Barton S, Navarro V, Robson JL, Wright MJ, Goodship JA (2007). Sequencing EVC and EVC2 identifies mutations in two-thirds of Ellis-van Creveld syndrome patients. Hum Genet.

[B30] Yang S, Langer L, Cacciarelli A, Dahms B, Unger E, Roskamp J (1987). Three conditions in neonatal asphyxiating thoracic dysplasia (Jeune) and short rib-polydactyly syndrome spectrum: a clinicopathologic study. Am J Med Genet.

[B31] Elcioglu N, Hall C (2002). Diagnostic dilemmas in the short rib-polydactyly syndrome group. Am J Med Genet.

[B32] Takamine Y, Krejci P, Wilcox W (2004). Mutations in the EVC1 gene are not a common finding in the Ellis-van Creveld and Short-Rib-polydactyly type III syndromes. Am J Med Genet.

[B33] Maroteaux P, Savart P (1964). La dystrophie thoracique asphyxiante. Etude radiologique et rapports avec le syndrome d'Ellis-van Creveld. Ann Radiol.

[B34] Kolowski K, Szmgiel C, Barylak A, Stopyrowa M (1972). Difficulties in differentiation between chondroectodermal dysplasia (Ellis-van Creveld syndrome) and Asphyxiating Thoracic Dystrophy. Aust Radiol.

[B35] Stone D, Slavotinek A, Bouffard G, Banerjee-Basu S, Baxevanis A, Barr M, Biesecker L (2000). Mutation of a gene encoding a putative chaperonin causes McKusick-Kaufman syndrome. Nature Genet.

[B36] Pirazzoli P, Mazzanti L, Mandini M, Cau M, Ravagli L, Cacciari E (1989). GH-deficiency in Ellis-van-Crefeld Syndrome: response to remplacement therapy. Growth Abnormalities.

[B37] Shibata T, Kawabata H, Yasui N, Nakahara H, Hirabayashi S, Nakase T (1999). Correction of knee deformity in patients with Ellis-van Creveld syndrome. J Pediatr Orthop B.

